# Awareness of dementia and coping to preserve quality of life: a five-year longitudinal narrative study

**DOI:** 10.1080/17482631.2020.1798711

**Published:** 2020-08-11

**Authors:** Kirsten Thorsen, Marcia C. N. Dourado, Aud Johannessen

**Affiliations:** a Norwegian National Advisory Unit on Ageing and Health, Vestfold Hospital Trust, Tonsberg, Norway; b Norwegian Social Research (NOVA), Oslo Metropolitan University, Oslo, Norway; c Center for Alzheimer’s Disease and Related Disorders, Institute of Psychiatry, Universidade Federal do Rio de Janeiro, Rio de Janeiro, Brazil; d Department of Nursing and Health, Faculty of Health and Social Sciences, University of South-Eastern Norway, Norway

**Keywords:** Awareness, coping, dementia, young-onset dementia, longitudinal, qualitative narrative study, quality of life

## Abstract

**Purpose:**

To examine how people (<65 years) with young-onset dementia (YOD) express awareness of dementia and how they seem to handle awareness as a strategy to preserve quality of life over time.

**Method:**

A longitudinal qualitative study with individuals with YOD was performed with interviews every 6 months over 5 years for a maximum of 10 interviews. The interviews were analysed by modified grounded theory adapted to narrative inquiry.

**Results:**

Awareness is a complex, multidimensional concept. Awareness of dementia is predisposed by personality, life history and established coping styles. The main coping styles during dementia—live in the moment, ignore the dementia, and make the best of it—seem to be rather consistent throughout disease progression. Transitions in the life situation may change the individual’s awareness of dementia.

**Conclusion:**

Unawareness of dementia may have an important adaptive function for preserving quality of life. Increasing awareness of dementia must be approached with reflexivity and great sensitivity.

## Introduction

Awareness in dementia has stimulated research for years, mostly cross-sectional, examining the individual’s recognition of difficulties associated with dementia at one point in time. The predicted increase of dementia worldwide (World Health Organization [WHO], 2017) has maintained this continued interest, but there is a scarcity of longitudinal studies examining the development of dementia awareness over time.

Dementia is a broad category of chronic, progressive brain diseases that reduce cognitive abilities and functional capacities. The self is threatened. No treatment is available for these brain diseases, but some medical treatments may temporarily improve or delay symptoms. Non-pharmacological interventions can alleviate the status of the disease and the situation and improve quality of life. Ordinarily, the disease is conceived as one that affects mostly older people (Prince et al., 2013), but it may develop before the age of 65 years. Often, it is then referred to as young-onset dementia (YOD). The number of people with YOD in Norway is estimated to be 4500–5000 (Engedal & Laks, 2017; Prince et al., 2013; Zhu et al., 2015) compared to older people with dementia, calculated to be 80 000.

Impaired awareness of memory loss is reported as a typical characteristic of Alzheimer’s disease, also in its early stages (Clare et al., 2016; Cosentino & Stern, 2005; Markova et al., 2014; Sousa et al., 2018, 2015). Applied to dementia, one definition is: “Awareness of disease in dementia is the ability to recognize deficits and cognitive impairments related to the disease process” (Dourado et al., 2014). The definition underlines the person’s awareness of deficits and cognitive impairments attributed to and, presumably, caused by the dementia process. A broadening of awareness of dementia was launched by Trindade et al. (2019, p. 1292-1299), who pointed out that it comprises “a person’s recognition of his or her general condition, and the associated impairment may be expressed in diverse ways”. Awareness of the dementia disease includes the ability to monitor one’s actions, make judgements and reflect upon the cause of impairment and how the condition affects individuals and their interactions with the environment (Clare, 2003). This cognitive task of awareness is complicated and is related to different types of dementia and updated medical definitions as well as the information presented—eventually by various health-care personnel—and understood by the person with dementia. It will also be influenced by cultural norms for the disease and stigmas attached to it (Johannessen et al., 2018; Rose, 2003; Voris et al., 2009).

Awareness is always attention focused on “something” (Clare, 2002); this has been called a particular “object” and is, presumably, “situationally specific” (Markova & Berrios, 2014). Depending on the “object”, awareness may include a broad range of reactions, which may include internal states, the self, mental representations, cognitive symptoms, functional impairments or changes in behaviour or relationships (Lacerda et al., 2016). Emotional reactions were added by Starkstein (2014).

There is a recognition that awareness varies across “domains” of functioning (Clare et al., 2016; Markova et al., 2014). Sousa et al. (2015) pointed out that awareness is different for cognitive and functional aspects in mild Alzheimer`s disease. Awareness in dementia will also be influenced by personality traits, coping style, and past experiences (Clare & Pratt, 2005) as well as the stage of the dementia (Clare, 2003; Clare & Wilson, 2006). This very broad range of reactions, in different domains (Starkstein, 2014), represents challenges for measuring awareness in dementia. Clare (2002) proposed that an apparent degree of unawareness might be an expression of an *adaptive* psychological response constructed in social situations in the context of neurologically based cognitive impairments. Her suggestion of an adaptive psychological response opens up perspectives of *positive* functions of unawareness. She pointed out that the evaluation of awareness is constructed in dialogues through the interpretive activity of the researcher/clinician. The same will be the case with evaluations or constructions provided by family carers as informants. Awareness is, thus, a highly complex phenomenon—assessed by the individual’s presentation and willingness to disclose his or her reactions in the dialogue with the interviewer. Expressed awareness may be “partial, intermittent or fluctuating” (Clare, 2002, p. 296). Clare also noted that there may be a dissociation between explicit verbal expression and a behavioural response, indicating awareness at an implicit or unconscious level (Weinstein, 1991).

Researchers have made different analytical distinctions of awareness. Markova (1997) pointed to *general awareness of change, awareness of a specific symptom*, and *awareness of the implications of the symptom*. Clare (2000), concentrating on reactions, distinguished five domains relevant to awareness: *registering changes, reacting to changes, finding explanations for changes, experiencing the impact of changes*, and *adjusting to changes*. These domains may be stages in the coping or adaptation process. Another version of conceptualization focuses on the time dimension: recognizing *what is happening now, emergent awareness*, and *anticipatory awareness*, that is, foreseeing what will come (Clare, 2002; Crosson et al., 1989). Recognizing what is happening at the present time presupposes comparisons with a former state, i.e., with how it was before. This is explicitly formulated in the Psychosocial Impact of the Diagnosis of Dementia scale (ASPIDD) (Dourado et al., 2014), which inquires whether the person is sadder, more anxious and angrier than before. Assessing change is the core of awareness of dementia disease. Clare (2000, 2002) related the functions of coping with awareness of the self in a continuum from *self-protective* to *integrative*, where self-protective responses are those that support the pre-existing sense of self and retain continuity, while integrative responses serve to integrate the development of dementia into the self-concept. Awareness studies have to consider psychological and social factors as well as biological changes. Reactions may be understandable as psychological coping efforts to avoid depression and preserve quality of life during the progression of the disease.

A general result from the few longitudinal studies of awareness of dementia is that self-ratings of people over a period of about 1 year were rather stable (Clare & Wilson, 2006). Several studies indicated that there may be differences in awareness among people with YOD and those with late-onset dementia (LOD). Functional status predicted awareness in LOD (Alzheimer’s disease) but not in YOD (Baptista et al., 2019; Dourado et al., 2016), while people with YOD were more aware of the disease than those with LOD (Baptista et al., 2019). In the younger group, preserved awareness was related to the worse self-reported quality of life (QoL).

Dementia is a disease with an uncontrollable outcome. It is well documented that preserved awareness of dementia and its symptoms is related to depression (Conde-Sala et al., 2013; Horning et al., 2014; Mograbi & Morris, 2014). Cines et al. (2015) examined the pathways between self-awareness, well-being and depression among persons with mild to moderate Alzheimer’s disease. They identified a direct association between awareness of memory deficits and depressed mood but not between awareness and quality of life. However, there was an indirect association, and this was through depression. Thus, for people with dementia, the way to preserve quality of life will be to reduce and avoid depression with various coping strategies. They may psychologically try to adapt and minimize the psychological impact of the depressing aspects and prospects of having dementia.

The present article is based on a narrative study to simulate rich, open personal stories about experiences of living for years with dementia, a somewhat uncharted research field. As underlined by Chase (2011, p. 421), *narrative inquiry* is a particular type of qualitative method inquiry and may be referred to as a subtype. It is based on an interest in life experiences as narrated by those who live them. A narrative is a distinct form of discourse, a way of understanding one’s own and others’ actions, of organizing events and objects into a meaningful whole, and of connecting and seeing the consequences of actions and events over time. People’s wording of their experiences relates to important nuances in meaning. The contexts to which they relate their experiences, the emotional tone and the intensity of the feelings all provide information about the development of their quality of life. We find the narrative method most suitable for this study compared to other qualitative approaches, which, to a larger degree, fragment texts into units and categories (Josselson, 2011). Additionally, the narrative method invites rich, individual accounts of meaning-of-life experiences (Chase, 2011).

Qualitative studies of the narratives of people with dementia have convincingly documented that they are able to talk about their reactions, reflect on them and give detailed introspective and complex reports about living with dementia (Clare, 2002; Johannessen et al., 2019; Thorsen et al., 2018; Trindade et al., 2019). To our knowledge, no other study has explored awareness of dementia based on people’s narratives of their experiences over such a long period—here, 5 years. With this background, we have studied the narratives of ten people with YOD in a longitudinal study over 5 years. We have analysed in detail the development of awareness of dementia over time concentrating on two cases, a man and a woman, combined with analysis of the total group of participants.

Aim: To examine how people with YOD express awareness of dementia and how, over time, they seem to handle awareness as a strategy to preserve quality of life.

## Methods

### Study design

This longitudinal, narrative study was conducted over the course of about 5 years. The first interview was conducted on 1 November 2014; the final interviews with the three remaining participants took place in November 2019, covering a total of 61 months. Individual, face-to-face qualitative interviews were conducted every 6 months with a series of up to 10 interviews with each participant. The first interview took place at inclusion in 2014, three to 6 months after the participant had received his or her diagnosis. A period of about 6 months between subsequent interviews was considered optimal, covering experiences of significant transitions during the interviews but not so frequent as to overtax the energy and willingness of the individuals to participate. An important consideration was to preserve trust and positive relations with the participants and to avoid exhausting them. Even at the tenth visit, the researcher (AJ) was warmly welcomed; “I can discuss more with you than with the family” was one comment indicative of this. All interviews covered what the participant experienced on the day of the interview and his or her earlier experiences and reactions to living with dementia since the previous interview. Sometimes, earlier themes were also referred to and discussed.

### Participants

To attain heterogeneity, we included persons of both genders with YOD living alone (age at onset of dementia younger than 65 years of age) and from the southern and western parts of Norway. Six memory clinics at six hospitals where the diagnostic assessments took place recruited the participants, who were invited to take part in the study when they came for follow-up examination 3 months after the diagnosis was decided. The participants had different dementia diagnoses (mostly Alzheimer’s disease) and various comorbidities. An exclusion criterion was frontotemporal dementia since people with this diagnosis have less insight into their disease and situation (Bott et al., 2014). In all, 10 persons were asked to participate, and none declined. The initial sample comprised participants 49 to 67 years of age (mean age 60 years) including 7 women and 3 men. By the time of the final follow-up interview, six persons had dropped out; one had died; one had developed severe dementia and was no longer able to answer the interviewer’s questions; three declined further interviews; and one was excluded because of the value of the information collected at the previous interview. Eight participants were divorced; one was a widow; and one was unmarried. Only 3 of the 10 participants had a child or children living close by but not in the same household. Further information is provided in Table I.

**Table I.** Characteristics of the informants, their living situation/residence and use of day care centre during the series of interviews from 1 to 10.

	1	2	3	4	5	6	7	8	9	10
Gender (age)	Residence	Residence	Residence	Residence	Residence	Residence	Residence	Residence	Residence	Residence
A: W	Nursing home^a^	Home	Home	Nursing home	Nursing home	Nursing home	Nursing home	Nursing home	Nursing home	Nursing home
B: W	Home	Nursing home	Nursing home	Nursing home	Nursing home	Nursing home	Nursing home	Dropout		
C: W	Home	Home	Home	Home	Home	Supported living ^c^	Supported living	Supported living	Supported living	Supported living
D: W	Home	Drop out								
E: W	Home Day care centre	Home Day care centre	Home Day care centre	Home Day care centre	Home Day care centre	Home Day care centre	Nursing home Day care centre	Dropout		
F: W	Home	Home	Home	Dropout						
G: W	Home Day care centre	Home	Home	Home	Home	Supported living	Supported living	Supported living	Supported living	Supported living
H: M	Home	Home	Nursing home	Nursing home	Nursing home	Nursing home	Nursing home	Nursing home	Nursing home	Nursing home
I: M	Home	Home	Home Day care centre	Dropout						
J: M	Home	Home	Home	Home	Home	Home	Home	Dropout^b^		

^a^Living in a nursing home shortly before moving to own flat; ^b^ Not possible to communicate anymore/little information; ^c^ Supported living accommodation (Supported living). Gender: Woman = W; Man = M.

During the follow-up period, two persons had moved to apartments in a supported-living complex; one continued to live at home but had respite stay in a nursing home; and three had moved to a nursing home. By the time of the tenth interview, the remaining participants—one man and three women—were all living in a nursing home or an apartment in a supported-living complex (see Table I). In this study, *supported-living accommodation* refers to a private flat in a complex of flats organized for people who require more assistance than those living in traditional flats. There, residents may individually receive varied types and levels of support, but the complex is not fully staffed 24/7 as would be the case for *a nursing home*.

For a more detailed analysis, we have analytically chosen two cases, Anne and Arne, among the four people remaining at the tenth interview. The two were those who most clearly demonstrated quite different styles of coping and awareness of dementia. Case study made possible a more individualized and contextual demonstration of the development of awareness over time. These two participants had provided rich narrative material concerning their views on the development of dementia, coping strategies and quality of life. The presentation of these cases is in accordance with the holistic narrative analysis. Thereafter, in order to present nuances and variations, we analysed and compared their results with those for the whole group.

### The interviews

The interviews were conducted as dialogues. A total of 68 interviews lasted for 7 to 63 minutes each (mean 28 minutes), and the total time for all the interviews was 1,906 minutes. The duration of each one depended on the individual respondent’s personality, verbal style and interview situation as well as the stage of the disease. Some interviews were rather short; the respondents’ styles were not as verbal and narrative but more matter of fact in their descriptions. This may represent a habitual sociolect (Uri, 2018). Sometimes the interview was disturbed or interrupted, or the participant did not feel well. The participants were in different stages of dementia when the assessment procedures began and the diagnosis was determined. Typically, participants’ narratives were shorter in later interviews. Verbal fluency and vocabulary seemed to be variously reduced during the progression of dementia.

The last (author) conducted all the interviews at the location that was most convenient for each participant (Denzin & Lincoln, 2011). That the same researcher was able to conduct each interview over time was a great asset for the continuation of the project. The interviewer established trust and open communication in line with the recommendation (Solomon et al., 2019) for longitudinal qualitative analysis. Establishing good rapport is fundamental for continued voluntary participation in a longitudinal study over a long period. Two of the interviews were conducted at a hospital; 7 took place at flats with supported-living accommodations; and 13 were conducted in a nursing home. All the other interviews were conducted in the participants’ private homes. Field notes were taken sporadically to supplement memory and interpretation, but the basic data material is the transcribed interviews with each participant’s own wording. The interviews were tape-recorded and transcribed verbatim by a professional typist within 2 weeks. The interviewer, AJ, performed quality control by listening to the tapes while reading the interviews. That process was sufficient and satisfactory since the quality check was to determine whether the research assistant had understood and transcribed the interviews accurately. The permanent interviewer was the person who could best understand what was said during each interview. The interview guide is shown in Table II; the first question in Table II was asked only at the first interview. Detailed follow-up questions explored what had happened since the previous interview.

**Table II.** Questions and themes from the interviews with the people with younger-onset dementia living alone.

Can you describe how it all started, the changes, and the process of being diagnosed (asked only at the first interview)?
How is your everyday life since you received the dementia diagnosis?
Can you describe the changes you have experienced and how you cope with them?
Does the disorder affect your relationships or contacts with family members or other people?
Do you feel that you are included in the treatment and services you receive?
Have you experienced other people making decisions for you? If so, how does that make you feel?
Has your quality of life changed since receiving the dementia diagnosis?
Do you need support or information of any kind?

### Analytic method

For analysis, we have used a *reformulated* analytical method approach of grounded theory (Corbin & Strauss, 2008). The method has been applied, without an intention of formulating a theory, to acquire more detailed knowledge about how people with YOD experience their life situation. As underlined by Corbin and Strauss (2008), examining a rather uncharted field without an intent of theory building is a necessary and valid scientific endeavour. The modified method is particularly fruitful for the study of people’s lived experiences of personal development and their social relationships, making it the preferred method for this study.

At first, an initial approach was to read all the interviews with an open mind whilst searching for accounts and remarks concerning the participants’ significant experiences and approaches related to awareness. Indicative sentences and comments were marked. Solomon et al. (2019) recommended both inductive and deductive interpretation for longitudinal analyses of large quantities of qualitative data, which we applied. The two authors (KT and AJ) performed the empirical analysis of the interviews (in Norwegian). We explored these significant themes “vertically” over time for each individual. This analytical approach is in line with holistic analysis for narrative inquiries. Josselson (2011, p. 226) pointed out that what is perhaps unique in narrative research is that ‘it endeavours to explore the whole account rather than fragmenting it into discursive units and thematic categories. It is not the parts that are significant in human life, but how the parts are integrated to create a whole, which is meaning’. Agreement on the significant approaches to awareness by the participants was reached by discussion among all the researchers. We also analysed it *horizontally*, comparing each participant’s situation and experiences with those of other participants in line with the method’s comparative approach (Corbin & Strauss, 2008). The two ways of analysing are intertwined empirically. Further, our analytical axis is *time.*

## Ethics

The study followed the ethical guidelines outlined in the revised Declaration of Helsinki (World Medical Association, 2013) and was approved by the Regional Committee for Ethics in Medical Research, Southern Norway (number 2013/2149). The Norwegian Data Protection Authority also approved the study (number 36797). The participants received oral and written information about the study and gave their written consent before they were interviewed. The names are fictitious.

## Findings

In the presentation of the empirical analysis, we will draw on the narratives of all participants, including those who left the project in the earlier phases and those who remained through the tenth interview (see Table I). We will first analyse in detail the stories of Anne and Arne, who participated in all 10 interviews. Then, as mentioned, the analysis of awareness for the whole group of participants will be presented.

### Anne

Anne is divorced. For more than 30 years, she has worked as a director of a commercial firm and has been engaged, busy, and hard-working. She has an adult daughter and two siblings, and her mother is still living. Her medical status is that she has three diagnoses and is on medication for all of them. At the first interview, she talked about how she became aware that something was wrong:

I understood. I went to the doctor in 2011. Then I had for a long time recognized that something was wrong, “Now I am about to get crazy!” Forgetting, remembering, suddenly being scared of things I have always done without any problems, like taking the train to the airport. Ah! It is a nightmare. So, I went to the doctor.

A number of tests were performed, and at the next visit, the doctor abruptly informed Anne that she had a diagnosis of Alzheimer’s disease. She described that the message came like “a BOMB”! Even though she realized that something was wrong, she had never thought of dementia: ‘I thought it was something that could be fixed, so it became a little … (…). When I did not know anything, I could just burst into anger, from frustration to getting furious. I, who never used to be angry. Now I have turned the anger towards myself’.

Initially, Anne received written information about dementia in the form of several leaflets and brochures. “When I came home, I quickly scanned them and got angry. This I shall …. This is rubbish! It is not true! No!” To realize the diagnosis in all its facets and contexts took time. She mentioned that she would rather have had cancer: “Either you die or get cured. It is either—or”. Anne knows that dementia is a deadly disease and wishes for it to develop fast. “Now it can be 10–12 years before you just tip over. You know nothing. Can’t plan anything. Nothing! I, who loved to travel!”

Being given a diagnosis of dementia was a blow to her everyday life and her activities, and a total break in her life course and life plans: “I had planned to work for nearly 20 years more. It will be difficult”. Her daughter had accompanied her to the doctor—a positive experience. Earlier, her daughter could become irritated with her mother’s problems. They also met the dementia team, a psychologist and a social worker who had knowledge and experience with dementia and were understanding and efficient in regard to the bureaucratic intricacies.

At the first interview, Anne is on sick leave from her job. She has informed a few of her colleagues about the diagnosis—“Those who can keep their tongue”—and she does not want her diagnosis to be spread. “They all say, ‘We don’t notice anything. Nothing at all!’ That is well enough, but I feel it so tremendously myself!” She recognizes her affected memory, her reduced abilities and emotional lability, and she knows the prognosis of her disease. She is, thus, fully aware of the signs and symptoms of dementia, even though it has taken some time.

At the next interview, she reports that her dementia contact has suggested that she joins a support group for dementia patients. She went to it once, but ‘Then I just went down. It was just too much for me. I was miserable for many days afterwards’. The social occasion and seeing persons with dementia more ill than she is reminded her of her grim future with dementia and broke her down. At this stage, she reports that her days feel empty and depressing, her existence meaningless.

Often, when I get up in the morning, I just return to bed. This is the worst, to get my days to pass, to get finished with one day after another. I can’t read any longer; I was a passionate reader. I just watch very simple programs on TV. Films are outside my reach. [I] Tried listening to books on CD, and they read too fast. I feel so stupid! Is it possible?

Anne says she postpones everything until tomorrow. She avoids meeting people, and social occasions get more and more difficult. “It is so hard to communicate. I use so much energy. And I feel I must use a lot of energy just to sit silent!” The future seems very frightening. ‘I have got a sort of panic. I do not know. I have written down what I want and do not want, the day I no longer can decide for myself. It rotates in my head’. Anne remarks that, should she develop another lethal illness, she will have no treatment. ‘What my body is unable to fix by itself, it just has to pass its way. Medicine? I just think: What is the point? Just to prolong the disease process? I do not want to be an empty shell’. She is adamant that she will decide for herself. She says that the information about dementia a doctor at the hospital gave her was incomprehensible.

I had incredible problems understanding what he said. I just fell down and have almost not been out afterwards. He even talked about the most basic level, the cells, and I thought: I do not have Alzheimer’s anyway! I did not understand a tenth of what he said. Not my daughter or my dementia coordinator either. She explains in a very good way, a normal way, not the medical language. She is very clever. I appreciate that very much.

The dementia has resulted in a total break with her identity, lifestyle, and level of activity. “What I think is most despairing is that I always have been a woman with a lot of energy. (…) And now: No energy at all. It is a great change. The sofa is very good [she laughs]”.

Both Anne’s mother and her daughter are very reluctant to take in information about the disease and listen to her experiences, while her brother is supportive. She feels in-between—not belonging to any group any longer. ‘I am not there (among people with dementia) yet. But when being with friends, I am not there either. So now, I am only just here (at home). I sleep very much’. She thinks other people see her as “incredibly lazy”. Her experience is that everything is distorted. She sees no future.

Anne begins the next interview by informing the interviewer that she has just “started on a new chapter”. She has decided to discontinue all her medication, even sleeping pills, and has refused all further medical interventions.

I have realized that to live with this [the dementia] is a hell, both for me and others. It is no fun. I feel I am much worse. People do not see it, but I feel it. So, I isolate myself more and more.

She has also ended all contact with the home nurse and her dementia contact—she “did not see the point”. “If I do something, it has to be with family and friends”.

From a lecture by a dementia specialist, she has learned that the mean survival time with dementia is 8 years. She is relieved by the information since “it is terrible not knowing”. Now she can count her time remaining and has started to “clean up”.

Sometimes, she experiences attacks of panic when she cannot find things. She reflects on how difficult it is to discern the causes of the changes she feels:

When I start to feel the changes in myself, it is hard. I get really depressed when I feel that this is this thing, and this and that. Now, I feel I do not know if it is the Alzheimer’s or whatever.

Her days are long and empty; she longs for the time when she can go to bed.

It has been suggested that Anne moves to a flat in a complex with supported living for people of all ages with varied disabilities, and she has accepted this suggestion. At the sixth interview, she has moved into supported living, and her mood and tone have changed completely. She talks eagerly about how she is decorating her new flat, with encouraging assistance from her mother and support contact. She enjoys shopping for furniture, curtains, and new gadgets. However, all the new appliances—washing machine, dishwasher, TV—are impossible challenges, and written instructions are of no use, although she gets assistance from personnel in the building. She talks about outings with friends to cinemas, concerts, and cafés. She has made plans to travel with her daughter: ‘We have so many plans. We would like to go to Africa’. Her everyday life brings her joy, and her future holds the hope and anticipation of pleasure. The researcher remarks that she seems to be thriving and doing better than before. She answers:

Yes, I am more satisfied. I am! I was more anxious in my former flat. Now I feel much safer. I have landed in a way. There are so many who ask, “Don’t you miss your former flat?” I say, “Not for a minute!”.

Anne praises the kind and considerate personnel at the complex. She summarizes the changes: ‘Yes. I thrive better and better and better. I really do, although I sense that I have deteriorated. Then I get very annoyed’. She persists in avoiding people from the dementia team; they cause her to feel much worse. She states, “All my life, I have said yes. Now I will end that. Learn to say no!” Bidding farewell to the researcher, she remarks, “I think it was a very good thing that I moved while I was as well as I am. Then I can get new habits and adapt to everything”.

The final interview with Anne focuses mostly on everyday events, joys, and minor practical problems. In her new social environment, with people all around, she looks forward to being alone:

It is very important to have time for myself. I am never bored in my own company. I think it is wonderful! Eat when I want, sleep when I want, go walking when I want, and not when I don’t want.

Her depression and anxiety and bouts of panic have vanished. But even in her life now, filled with pleasures and plans, a continuing stream in her existential experience is the awareness of her deterioration due to the disease:

I am tired of being ill. I am fed up. I can get furious because I have heard that it takes 5 to 12 years. Then I think, when 12 years have passed, maybe I will be relieved of all this. Finished. I am not afraid of dying. I have planned. When I am leaving, everything shall be in order!

### Arne

Arne is divorced, has two adult sons, and is living alone in a remote part of the country. His opening remark at the first interview is that he is now “unemployed” from his work as a leader of a department with many employees in a large firm. At a meeting with the company’s director, he was informed that he seemed to have trouble concentrating and then quickly passed into sick leave. He does not mention anything about a medical assessment or a diagnosis and has no feeling that anything is wrong. There was much to keep track of in his job, he says, and he is not really motivated any longer. However, he does mention that his memory is not what it used to be. When asked, he answers that he has all the medical information that he needs. The word “dementia” is not used. His concern now is to try to look forward and find new activities to fill the void from his job. He is reading a lot. Then, his narrative turns into a lively, very detailed life story about his education and working life, bringing him all over the world: “It has been an unbelievably rich experience”.

When he is asked if he has talked about his illness with his family, he assumes that they know. “To be clear, I do not talk much about illness in general. I imagine that I may become more ill by that, but I don’t know”. He says he has never had many friends. Two home nurses are brightening his days, and he has visits from home helpers every day. This assistance is evidence that he needs—and also has—rather much help for a dementia disease he has not mentioned with the word “dementia”.

At the opening of the next interview, he presents his main coping strategy: “I have really no reason to complain about anything”. Then, the researcher asks Arne directly if he notices any development of the dementia. His concise answer is that he does not. He thrives very well in his house. He still has his driving licence and foresees that losing it will be a crisis. Later on, the researcher directly poses the question: “Do you think much about the illness?” Arne’s answer is the same; he does not, and he emphasizes that life is good. “It is OK, having full freedom, with full salary”. When asked about his quality of life, he does not feel that it is reduced. Just before leaving, the researcher makes a last effort to attempt to get Arne to comment on experiences of his memory. He persists in saying that he has no experience of changed memory or forgetting more frequently. He is as he always has been. He presents a persistent approach of non-awareness.

At the third interview, Arne has moved into a nursing home. His family doctor suggested an ambulatory (relief) stay, which became permanent. He felt that he managed okay, “But it depends on who is looking”. However, now he has got something to complain about. After having praised the food, the service, and the personnel, he says: “But I have lost my freedom!” He continues to say that he does not experience reduced memory: “Maybe because I am stupid. However, I remember like an eagle what happened 15 years ago”. He has sold his car and finds his days empty and terribly long: “17 hours awake here, if you have nothing to do. (…). I am longing [for] home really. However, I do not lie awake and cry”. He is unaware of why he came to the nursing home. “I have to admit that I do not know what happened. My doctor meant that I could need to be looked after”. He wants “to get out”.

Arne finds that there is a clear link between negative thinking and negative feelings. ‘I have realized that to go and sense such things, then it quickly becomes negative. So, we have to take one day after another and whatever comes’. He adheres to this position of non-attention to the dementia throughout the later interviews, his chosen strategy for coping. When asked how he experienced “the disease”, he has no special experience of having Alzheimer’s disease: “The more you think about it, the sicker and sicker you get”. His reduction in quality of life is seen as caused by his lack of freedom. “You will never get your freedom back, which reduces quality of life. However, I am lazy. It is very convenient and comfortable here. I do not have to decide anything. Long live laziness!”

Arne thinks that it was a mistake to sell his car and lose his freedom. The thought that he was no longer *able* to drive does not seem to come to his mind. But he no longer would like to return to his house in the woods and says, “I will no longer live there”. Tactfully, the researcher attempts once more to focus the dialogue on Arne’s dementia: “Can you tell me what illness made you come here?” He mentions that it was blood pressure that brought him to be assessed at the hospital. “But you can read it (my medical health record); I have not sought it out. I am not very bothered by dementia, but I have *a hint* of it, so to say!” When asked more about this, he continues: “I do not experience it at all. Sometimes you can think, ‘Why did I do that?’” He does not see, or admit, or connect any problems to dementia. He says he can be a little “slow”, but it has always been like that. He has plans to get a PC, a desk, a printer and a musical instrument. At the sixth interview, he is no longer reading since he has “lost his glasses”. He socializes more with his neighbours and feels he has a positive social role. He repeats his slogan: “I see no reason to complain!” He still looks at the positive aspects of life.

At the final interview, Arne’s mood has changed: “I survive. But I thought that I should tell you that I have registered that I mix up a little, and then I get annoyed”. This is his first glimpse of awareness and admittance that “something is wrong with him”. The interview is short and his sentences shorter than before. He is also extremely tired and falls asleep several times during the interview. He seems to have come to a halt.

### Awareness among the other participants

We found that most of the participants in the study rather quickly (within at least 6 months of the first interview) following the initial shock of being diagnosed with dementia attempted to cope with the implications of the disease. Acceptance of the disease took some time, varying from quickly taking a stoic stance—accepting what life gives them, both the good and the bad—to despair and denial. However, all needed some time to absorb and react to the many consequences in different life spheres and to eventually attribute them to dementia.

The few who were still working and those who had rather recently taken a disability pension talked about the serious existential blow that it was to be informed that dementia had destroyed their working capabilities, to be placed on sick leave, and then to lose their job. To receive this diagnosis turned their daily lives upside down in every aspect, losing daily structure and routine, content in their day, status and position, identity and self-respect. Being forced to leave work left a great void in their lives. Adding to their difficulties was their vulnerable status as single. There was no one at home to give them attention and support or to share everyday life and, eventually, the conflicts dementia might bring. Two participants mentioned that they had divorced because their partner did not accept their new persona with dementia.

A typical strategy was to conceal the diagnosis from others and, if necessary, in awkward situations, to eventually hint at some memory problems. One man states, “I try to conceal it, and I manage, but my state is not good”. He appreciates his many good friends who look after him discreetly because of his reduced memory. He remarks that the only time he reflects on his disease is when the researcher returns for another interview. His non-awareness of the disease is continued during the entire project period. Later on, he even disregards his memory difficulties, declaring, “I have no problems!”

He and others mention that they *decided* to look at all the good things in life, to use this as a strategy and to appreciate them. They take an optimistic outlook. Several also mention an optimistic approach as a personal trait. People are *ageing* during the project period, growing into the category of “old people”, where reduced memory is considered a characteristic. The eldest participant mentions that her son comforts her by saying, “You are old, mother”, when she mentions her reduced memory. The man who regrets losing his job sees that his friends are also retiring. Then, he is returning to a normalized life course: “Suddenly, I am a member of the club again”.

A woman living in the remote countryside for the entire study period stresses that she lives a normal life as if this is how it is for most people. She normalizes her situation and presents non-awareness as an approach. Sometimes, life is a little boring, but it is for others as well. In the later interviews, she is visiting a day-care centre three times a week and enjoys the social life there. At the last interview, she also has an intermittent respite stay at a nursing home. Even so, she has no feeling that things are changing and says, “It is the same”. Her home represents continuity in everyday life, and public support makes for a better life—a supported life, a social life. She finds that “life is good, really”. At the final interview, both respondents who continued to live at home have the experience that they remember even a little better.

The most-decisive shift in awareness of the disease occurred among the two most-depressed, frightened and lonely respondents living alone who moved into supported living or a nursing home. In these settings, where they were seen and respected as individuals *with* dementia, the awareness of the disease was reduced, as it was for Anne. Their attention was turned towards vitalizing and enjoyable social activities.

All respondents attempted to adhere to the strategy of not looking ahead and avoiding awareness of the future. When the dementia developed further, the handicaps could make it more difficult to participate in social activities. One woman, no longer able to take part in social gatherings in the nursing home, missed personal contact and blamed the personnel for neglecting her. She became lonely once again, as she had been at home. The awareness of dementia again came to the forefront.

## Discussion

Awareness is not just a symptom of the dementia disease, which increases in severity. Our study underlines the significant influence of psychological and social factors on the individual’s awareness or lack of awareness of dementia, especially related to the person’s coping efforts. A main result is that unawareness is a prominent strategy when living with YOD to preserve quality of life and avoid depression. While other qualitative studies of awareness have reported reactions relevant for awareness of dementia at a specific time (Clare, 2000, 2002; Trindade et al., 2019), we have examined the participants’ narratives about existential living with dementia for a long time. The study has demonstrated that the individual’s *main* way of handling awareness, his or her coping style, has been rather consistent over time. Relevant for awareness—or more precisely—the admittance of awareness, is personality, preferred coping styles and health beliefs, in line with Clare (2002).

We find that preserving the self is the essential aim of coping strategies related to awareness of dementia when the disease threatens the self. “To be, or not to be” (Johannessen et al., 2018) denotes the pivotal role of the struggle and support for the self and self-respect among people with dementia. The core of the coping efforts is to support the self, to protect one’s dignity, to preserve quality of life and to avoid depression.

The participants for the present study talked about several ways of coping by handling awareness. A strategy such as *minimization* of awareness of memory problems may intend to reduce the problems and allow the person to “keep going” without becoming depressed by failure (Clare, 2002). Attributing difficulties to the *ageing* process and normal changes related to growing older reduces the impact of failing memory to what all older people experience (Johannessen et al., 2019). Other attributions in our study are *situational factors* such as losing one’s job and/or being given fewer challenges, *changes in mood, being lazy, lacking motivation, selective comparisons* with those considered worse off or *normalizing*—seeing life as normal and like others’ lives. These strategies are part of the ordinary coping repertoire of most ageing people in everyday life and may have been used for other purposes earlier in life. They are included in the main strategies people use for successful ageing, such as selective optimization with compensation (SOC) presented in the model by Baltes and Baltes (1990) and further developed by Baltes and Carstensen (2003). During ageing, people change their life goals (Brandtstädter et al., 2010).

Awareness is a construct, and an assessment of awareness takes place and is expressed in dialogues—with medical/psychological specialists, researchers and family carers—reacting to and reporting on the adequacy of the person’s reactions and awareness of his or her disease. Several studies use comparisons between the individual’s self-ratings and those by the carers/professionals (Baptista et al., 2016; Clare et al., 2016; Clare & Wilson, 2006; Dourado et al., 2016). The expression and registration of awareness derive from the interpretations made by the carer, the clinician, or the researcher based on the expressions or statements of the person being interviewed or assessed (Markova, 1997). Clare (2002) emphasized that *reflexivity* must be central to the research endeavour in attempting to measure and evaluate awareness. In empirical research of awareness, there is a challenge: Who is making the “correct” assessment of the person’s awareness of the disease and its influence on the general state of functioning? What professional or relational perspectives, or biases, interfere with their interpretations? Clare claimed that “Labelling unawareness as a ‘symptom’ results from a stance that distances researcher from researched and that objectifies the person with dementia” (Clare, 2002).

Our study underlines that the assumption that awareness in simple and direct ways reflects the dementia disease or, more specifically, different types or degrees of dementia, has to be replaced by reflexivity on the complexities of the relationships between the person, the disease and the situation, as well as with the evaluators of the person’s awareness. Studies have revealed that the informants or professionals’ evaluations differ from the person’s experiences of quality of life (Bosboom et al., 2012; Dourado et al., 2016; Sousa et al., 2018; Schulz et al., 2013; Vogel et al., 2012). We have shown that awareness and reactions to awareness are also influenced by external events and transitions that change the person’s situation over time, e.g., moving into supported living or a nursing home. The environmental context and the nature of caregiving interactions influence the extent to which awareness is expressed, in line with Clare (2010). For participants living alone with a high level of awareness of dementia, their situation was accompanied by serious and escalating depression and anxiety. These reactions were markedly—even dramatically—reversed when the person moved to supported living or a nursing home. We have demonstrated how transitions from isolated living to understanding and supportive environments where the person is accepted as an individual *with* dementia can revitalize his or her outlook and quality of life. The new milieu with support lessens anxiety and depression. The improvement is brought forward, as the participants describe, not by specific psychological or medical treatment but by understanding and patient persons (private or professional) who socialize with, accept, and support the person. This is seen as assistance with the *integrative changes of the self* (Clare, 2002), a process whereby the person accepts a self-transformation due to dementia.

As we have shown, a lack of awareness may have positive consequences, such as living in the moment and looking at the positive aspects of one’s life. It provides a shield against depression. Bonnano (2004) has asked: Have we underestimated the human capacity to thrive after aversive events, like receiving a message of serious chronic diseases? Some significant persons are necessary, mirroring the person’s self-perception as a valued individual. Kohut (1985) emphasizes the vital importance of positive responses from others for developing and sustaining a robust self with self-respect and coping capacities.

Family members may play such a role, knowing the person’s former identity and history. However, families may not be the best “self-supporters” for people with dementia (Barca et al., 2014; Johannessen et al., 2015). Family carers have their own needs. As Anne described, some family members may need to remain unaware of the relative’s diagnosis or even deny it from time to time. This defensive non-awareness by family members may be more prominent when the person is younger (with YOD) and looks younger, and the disease is thwarting their desire to enjoy a long future with the person who has dementia (Johannessen et al., 2018; Thorsen et al., 2018).

What are the clinical implications of our findings? What might be the consequences of teaching and supporting awareness of the disease of dementia to the person with the disease? The consequences of reduced awareness and the prospects of increased awareness—which may be the clinical goal of assessment of non-awareness—are described by Cines et al. (2015, p. 1298): Disordered awareness makes the patient less likely to comply with treatment, increases care burden, and impairs decision-making capacity. Consequently, interventions to improve awareness of cognitive and functional deficits will have potential value.

Is this the case? We found that people need varying amounts of time to accept the diagnosis, consider the implications, and experience the consequences of dementia in different life arenas and in different time perspectives—for daily life and prospects. The diagnosis has an impact on all aspects of life. It can be very difficult to sort out the causes of disabilities and emotional reactions. The participants may wonder: Is it the disease or the changed life situation? Professional people may, as we have seen, not be very adept at presenting the information in a way that is understandable for the person with dementia. The unawareness may be based on informational deficiency. People of different professions in different arenas may have different versions of information. People with dementia may need “translators”–advisers who are able to explain the disease and its consequences in ways that are understandable and acceptable. Awareness and adaptations may take a long time.

While unawareness may be protective of the self-esteem and identity of the person with dementia, it may be a burden and a barrier for families attempting to get assistance and relief. A study has found that the characteristic lack of self-insight and awareness of illness among people with frontotemporal dementia represents a significant challenge—and sometimes a “disaster” for family members (Johannessen et al., 2017). This group was, as noted, excluded from our study. The symptoms may prolong the pre-diagnostic stage and, thereby, the length of time without treatment and assistance. Moreover, rigid non-awareness has impacts on many aspects of family interaction, stirring conflict, aggression, and victim-blaming, as well as everyday difficulties like economic problems and the loss of a driver’s licence (Johannessen et al., 2018). Family doctors may distrust information about symptoms from family members when the person with dementia appears to be functioning adequately, presents a normal façade and is lacking the typical memory reduction symptomatic of Alzheimer`s dementia. Health-care professionals need to know about the different types of dementia and their symptoms and must be able to take into consideration the different experiences and needs of the family members.

We have seen that a lack of awareness does not necessarily lead the person to refuse assistance. Even Arne, practising unawareness most persistently, *has* accepted help and support and moved to supported living. In total, awareness of dementia takes place in relations that are complex and sensitive for all involved—the person with dementia and his or her family. People whose aim is to increase awareness must ask: for what purposes? for whom? for the person with dementia, for the family, for the professionals or the health and welfare system? Finally, most important is the person with dementia. Lack of awareness must be addressed by providing information in a sensitive, appropriate, and supportive manner (Clare, 2010, 2002), and efforts to enhance awareness should be undertaken only when awareness is likely to improve the person’s quality of life (Clare, 2010). The approach should be based on the premise that accepting the disease in all its aspects will take time, and the amount of time varies among individuals and across situations. The knowledge that non-awareness may have important coping and protective functions for supporting quality of life makes reflexivity and sensitivity of utmost importance.

## Strengths and limitations

Strengths of this study include the extraordinarily long period of contact and the numerous dialogical interviews based on trust (Kvale & Brinkmann, 2009), adding rich material that provides insights into the experiences, reflections, and awareness of dementia by people with YOD and their relations to quality of life. The longitudinal approach provides insights into personal coping strategies, their variations or consistency over time, and the expressed intentions and functions of the coping efforts for the person. The *meanings* of non-awareness and awareness are disclosed. The study leaves open the question about reactions and awareness of dementia among people with late-onset dementia. Exploring the ways in which awareness develops over time offers important information about processes involved in awareness and their relationships to other factors. Further longitudinal studies are needed.

## Conclusion

Our study has demonstrated that awareness of dementia is a complex, multidimensional, multilayered concept related to cognitive, emotional, behavioural/instrumental and social functions in different domains of life. For a person to receive a diagnosis of progressive dementia is a devastating message that transforms all aspects of his or her life. One’s awareness of having dementia is predisposed by personality, life history and established coping styles, as well as the development of the dementia disease. The main coping strategies—living in the moment, ignoring the dementia, and making the best of the situation—seem to remain rather consistent throughout the progression of the disease. Transitions in life situation and support may change the individual’s awareness and acceptance of dementia and integrate awareness into a transformed self. Increasing the individual’s awareness has to be approached with the utmost sensitivity and support.
